# Environmental and cultural correlates of physical activity parenting practices among Latino parents with preschool-aged children: Niños Activos

**DOI:** 10.1186/1471-2458-14-707

**Published:** 2014-07-10

**Authors:** Teresia M O’Connor, Ester Cerin, Rebecca E Lee, Nathan Parker, Tzu-An Chen, Sheryl O Hughes, Jason A Mendoza, Tom Baranowski

**Affiliations:** 1 USDA/ARS Children’s Nutrition Research Center, Department of Pediatrics, Baylor College of Medicine, Houston, TX USA; 2 Academic General Pediatrics, Department of Pediatrics, Baylor College of Medicine, Houston, TX USA; 3 Institute of Human Performance, The University of Hong Kong, Hong Kong, SAR China; 4 Centre of Physical Activity and Nutrition Research, School of Exercise and Nutrition Sciences, Deakin University, Burwood, Australia; 5 Affiliation where this work was done: Texas Obesity Research Center, Department of Health & Human Performance, University of Houston, Houston, USA; 6 Present affiliation: College of Nursing and Health Innovation, Arizona State University, Phoenix, AZ, USA; 7 Affiliation where this work was done: USDA/ARS Children’s Nutrition Research Center, Baylor College of Medicine, Houston, TX USA; 8 Present affiliation: Division of General Pediatrics, Department of Pediatrics, University of Washington School of Medicine, and the Center for Child Health, Behavior and Development, Seattle Children’s Research Institute, Seattle, WA, USA

**Keywords:** Physical activity, Child, Parenting, Correlates, Environment, Neighborhood, Acculturation, Latino

## Abstract

**Background:**

Latino children are at high risk of becoming obese. Physical activity (PA) can help prevent obesity. Parents can influence children’s PA through parenting practices. This study aimed to examine the independent contributions of (1) sociodemographic, (2) cultural, (3) parent perceived environmental, and (4) objectively measured environmental factors, to PA parenting practices.

**Methods:**

A cross-sectional sample of Latino parents (n = 240) from Harris County, TX in 2011–2012 completed validated questionnaires to assess PA parenting practices, acculturation, familism, perception of their neighborhood environment, and demographics. Home addresses were mapped and linked to Census block-level crime and traffic data. Distance to the closest park was mapped by GIS. Regression models were built in a hierarchical step-wise fashion.

**Results:**

Combined models showed R^2^ of 6.8% to 38.9% for different parenting practices. Significant correlations included sociodemographic variables with having outdoor toys available, psychological control, and promotion of inactivity. Cultural factors correlated with PA safety concern practices. Perceived environmental attributes correlated with five of seven parenting practices, while objectively-measured environmental attributes did not significantly correlate with PA parenting practices.

**Conclusion:**

Interventions promoting PA among Latino preschoolers may need to address the social-ecological context in which families live to effectively promote PA parenting, especially parents’ perceptions of neighborhoods.

## Background

Latino children carry a disproportionate burden of the obesity epidemic, starting at a young age [1]. Physical activity (PA) was negatively associated with adiposity in children [2,3], supporting that PA plays an important role in pediatric obesity prevention. It is therefore imperative to identify modifiable factors that influence Latino preschool children’s PA. Parents are an important social influence that affect Latino preschoolers’ PA [4]. Parenting practices (context specific parent behaviors intended to influence their child’s behavior) [5] for PA were linked to children’s PA [6]. PA parenting practices to support children’s PA were positively associated with school aged children’s PA [7,8]. For preschool aged children, parental use of PA to reward and control behavior, and logistic support for PA were associated with objectively measured child MVPA [9]. In a sample of Latino parents of preschoolers (n = 85), psychological control and registering children for sports was significantly positively associated; while promoting screen time was negatively associated with children’s objectively measured moderate PA [10]. Latino parents of preschoolers reported using parenting practices that both encouraged and discouraged PA [11].

From a Social Ecological perspective [12-14], multiple social and environmental factors should influence behaviors such as PA parenting practices. A few studies have examined social ecological correlates for supportive PA parenting practices [15,16], but not for parenting practices that may discourage PA among preschoolers, nor specifically among Latino parents. Research found that characteristics of the neighborhood were associated with children’s weight status [3] and PA [17-23], but it has not been established whether neighborhood characteristics are also associated with PA parenting practices. This would provide a potential mechanism through which the neighborhood characteristics may influence children’s PA, especially among younger children when parents are most influential.

Culture may also influence PA parenting practices. Mexican American mothers had different beliefs about parenting than European American mothers during pregnancy, which was influenced by their level of acculturation [24]. Acculturation also influenced Latino parenting in other contexts [25,26], but it is not known if cultural factors influence Latino parenting practices specific to children’s PA.

Identifying correlates of PA parenting practices that fit within the Social Ecological framework [27,28] can help detect targets for future interventions and policy changes to promote greater use of parenting practices that encourage PA and to decrease the use of those that discourage PA. The aim of this study was to examine the contributions of socio-demographic, cultural, objectively-measured and parent perceived environmental factors to explain variation in parenting practices that encourage or discourage PA among Latino preschoolers.

## Methods

**Figure 1:**
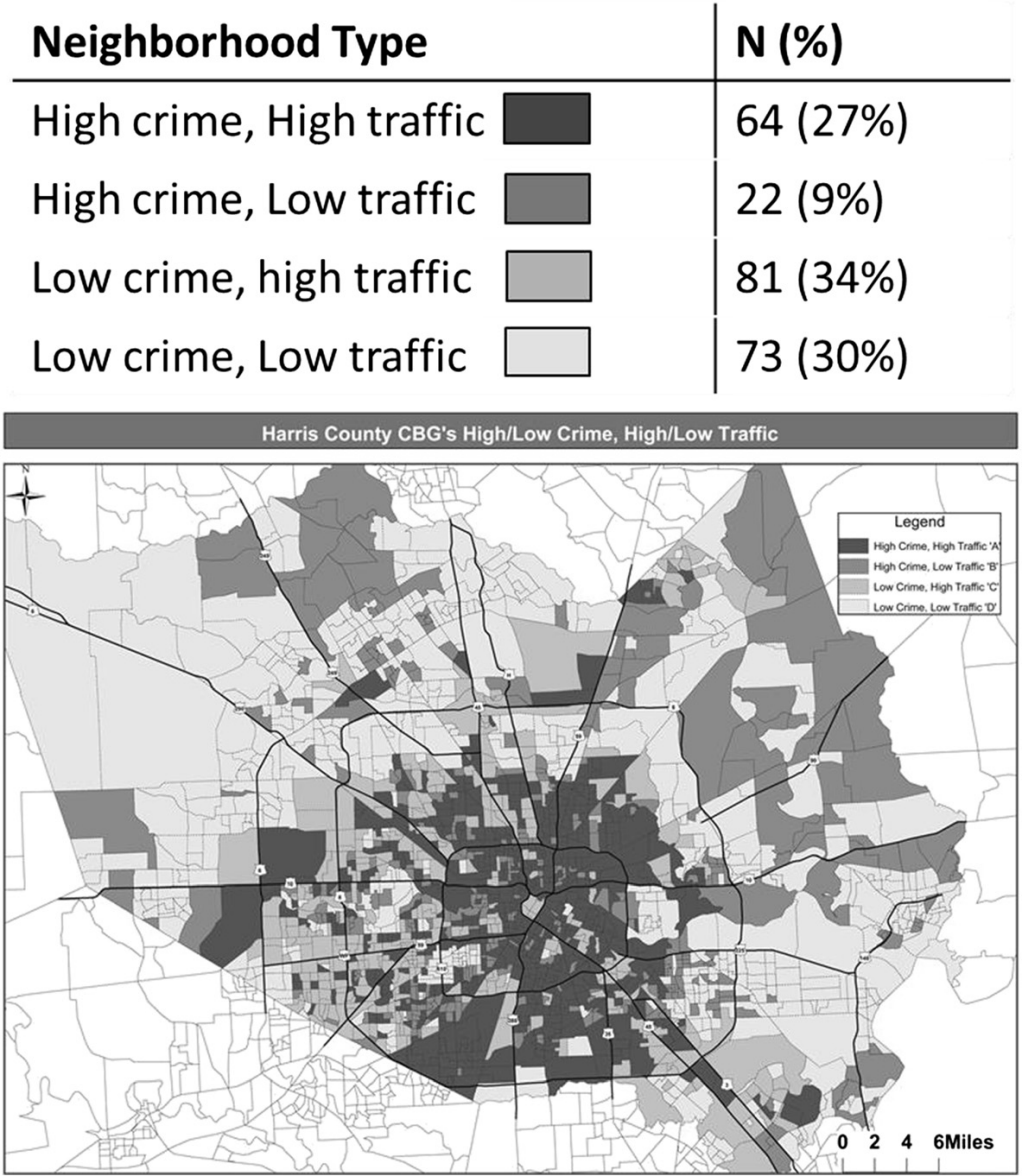
**Recruitment stratification of Niños Activos sample by traffic and crime risk in Harris County, TX (n = 240).** Recruitment occurred July 2011 to March 2012.

A cross-sectional study of Latino parents (n = 240), Niños Activos (“*Active Children*”), was conducted in Harris County (Houston), TX with data collected from July 2011 to March 2012. Data were analyzed in 2013. Details about the study have previously been reported [10], and are briefly described here. To ensure variability of the neighborhood environmental variables, parent recruitment was stratified by objective traffic and crime risk characteristics of families’ neighborhoods at the census block level [10]. Traffic risk indices were calculated based on traffic related injuries and motor vehicle miles traveled for each census block group in Harris County from 2004–2008 [10]. Crime index data, based on FBI Uniform Crime Report data from 1998–2007 at the census block group level for Harris County, were included (Tetrad Inc, Vancouver, British Columbia; http://www.tetrad.com) [10]. Using median splits of the crime and traffic safety index scores, each block group within Harris County was classified as high crime/high traffic; high crime/low traffic; low crime/high traffic; or low crime/low traffic. The goal was to enroll about 60 participants from each type of block group. Recruitment from census block groups with high crime and low traffic (typically in the outskirts of Harris County) proved difficult due to lack of infrastructure. After we maximized enrollment from the high crime and low traffic block groups and 60 participants were recruited for the three other types of block groups, we employed convenience sampling to achieve the goal of 240 total participants. Figure 1 depicts final recruitment stratification.

### Sample

Parents were recruited through various community organizations, events, and locations [10]. Inclusion and exclusion criteria were previously reported [10]. After providing informed written consent, parents provided their home address (for GIS mapping), and completed a demographic questionnaire and several self-report instruments to assess PA parenting practices, perceptions of physical- and social-neighborhood, and cultural-related factors. Participants received $20 in compensation for completing the questionnaires and in a sub-sample $20 if they completed surveys 2 weeks later. The study was approved by the Baylor College of Medicine Institutional Review Board.

Socio-demographic characteristics of the sample have previously been reported [10]. Most of the Latina respondents were mothers (95%); 52% reported their preschool aged child was male with a mean age of 4.5 (sd 0.9) years. Approximately 45% of the parents were born in the US, and 99% of the children were born in the US. Most reported family origin as Mexico (68.7%), followed by El Salvador (10.8%), and Honduras (4.2%). Nineteen percent reported English, 43% Spanish and 38% both as their primary language. Around 75% reported completing secondary education and 45% were employed. Approximately half (53.8%) reported living in a single family home or duplex. The majority (71.3%) reported their child spent some time in childcare, daycare, preschool or school every week.

### Measures

Prior to survey instrument use, cognitive interviews [29] were conducted with ten Latina parents, five in English and five in Spanish. This resulted in minor wording changes to a few of the scale items. Table 1 shows mean and median scores, along with the internal reliability (Cronbach’s alpha (α)) and test-retest reliability for each measure used in this sample.

**Table 1:** Descriptive statistics for cultural, objective and perceived environmental factors, and parental practice variables

**Variable (theoretical range of scale)**	**M (SD)**	**Median (IQR)**	Cronbach’s alpha (Average inter-item correlation)*	**Test-retest reliability (n = 48)**
***Cultural***				
Acculturation – Non-Hispanic (1–4)	2.8 (1.1)	3.1 (2.1)	0.98	0.64
Acculturation – Hispanic (1–4)	3.3 (0.7)	3.5 (0.9)	0.94	0.80
Familism (1–5)	4.6 (0.6)	5.0 (0.6)	0.90	0.82
***Objective environmental factor***				
Crime index	178.3 (125.6)	126.0 (195.0)	N/A	N/A
Traffic index (sum of z-scores)	1.1 (5.1)	-1.1 (6.2)	N/A	N/A
Distance to nearest park (km)	1.9 (2.6)	0.8 (1.5)	N/A	N/A
***Perceived environmental factor***				
Signs of physical and social disorder (1–5)	2.0 (0.7)	1.8 (1.0)	0.93^†^	0.95^†^
Traffic safety (1–4)	2.9 (0.7)	3.0 (0.7)	0.53 (0.28)	0.74
Traffic hazards (1–4)	2.7 (0.8)	2.8 (1.0)	0.80	0.63
Stranger danger (1–4)	2.6 (0.9)	2.6 (1.3)	0.93	0.78
Availability of active play equipment (8–32)	22.7 (5.7)	23.0 (8.0)	N/A	0.86
Places for child’s physical activity (0–12)	5.7 (2.9)	6.0 (4.0)	N/A	0.89
Neighborhood informal social control (1–5)	3.4 (0.7)	3.4 (0.8)	0.93	0.83
***Parenting practice related to physical activity***				
*Practices that Encourage child PA*				
Engagement/Structure (1–5)	3.4 (0.4)	3.3 (0.8)	0.90	0.85
Register child for sports or dance (1–5)	3.0 (1.4)	3.0 (2.0)	N/A	0.62
Have outdoor toys available for child (1–5)	3.9 (1.2)	4.0 (2.0)	N/A	0.57
*Practices that Discourage child PA*				
Safety concerns (1–5)	2.6 (0.9)	2.5 (1.8)	0.82	0.56
Psychological control (1–5)	2.1 (0.7)	2.0 (1.3)	0.59 (0.26)	0.85
Promote inactivity (1–5)	2.0 (0.7)	2.0 (1.3)	0.50 (0.26)	0.59
Promote screen time (1–5)	2.4 (0.7)	2.3 (0.7)	0.61 (0.34)	0.62

Note: M = mean; SD = standard deviation; IQR = interquartile range.

Data were collected in Harris County, TX from July 2011 to March 2012.

^†^Represents the modified 16 item for Signs of physical and social disorder scale. Cronbach’s alpha for the original 14-item scale was 0.93 and test-retest reliability was 0.94.

*Average inter-item correlation is reported for subscales with Cronbach’s alpha < .70 and 5 or fewer items.

### Physical activity parenting practices

Physical Activity Parenting Practices were assessed by the Preschooler’s Physical Activity Parenting Practices (PPAPP) instrument developed for this study (Table 1) [10]. The PPAPP consists of two scales: 1) parenting practices that *encourage* child PA which consist of a engagement/structure sub-scale (15 items), and two single-items (have outdoor toys; not enroll in sports-reverse coded); and 2) parenting practices that *discourage* child PA which include 4 subscales: promote inactive transport (3 items), promote screen time (3 items), psychological control (4 items) and safety concerns (4 items). Confirmatory factor analyses (CFA) found acceptable fit of the final models for both scales and some of the factors were correlated with children’s objectively measured PA [10].

### Cultural variables

Acculturation was measured using the Bidimensional Acculturation Scale for Hispanics [30] with 3 sub-scales: language-use, language proficiency and electronic media. As recommended [30], we combined the subscales to create a Hispanic domain and a non-Hispanic domain. The original study reported α = 0.90 and 0.96, respectively with appropriate validity coefficients for generation in US [30].

Familism is the cultural value of strong attachment and identification with one’s family. Among Latino groups, it often goes beyond a person’s nuclear family [31]. The Pan-Hispanic Familism Scale [31] consisting of five items was used. The original study reported an α = 0.82 with factorial invariance across language of survey and country of origin [31]. The average scale score was used in analyses.

### Objectively-assessed environmental variables

Crime and traffic risk were objectively assessed as described in the sampling method. We combined personal and property crime to create an un-weighted total crime risk for each census block and used this continuous variable in subsequent models. A traffic safety index was calculated as the sum of three principle-components traffic sub-factor scores expressed as z-scores [10] for each census block group for use in subsequent models. Participants were linked to crime and traffic risk at the census block group level by spatially joining their home address to 2000 Census TIGER/Line shapefiles for Harris County Census Block Groups using ArcGIS (version 9.3, ESRI, Redlands, CA). Shapefiles from 2000 were used since they corresponded to the data reported from Tetrad and Houston-Galveston Area Council (data collected prior to 2010). The distance in kilometers (km) to nearest park was calculated using the ArcGIS “Near” function to assess proximity between home addresses and all area public parks receiving formal maintenance.

### Parent-perceived environmental variables

Perceived signs of physical and social disorder were assessed using the ‘Disorder’ sub-scale from the Neighborhood Context scale [32]. The original sub-scale had an α = 0.95 [32]. We added two items (stray dogs; and public open spaces not kept up) which did not impact the α-values or test-retest reliability (Table 1). The mean scale score was used for analyses.

Perceived Traffic Safety was measured using an adaptation of the Neighborhood Environment Walkability Sale for Youth (NEWS-Y) [33] based on cognitive interviews and additions (e.g. “The majority of the streets have sidewalks that I can easily use.”). A CFA demonstrated good model fit [Yuan-Bentler residual-based *Χ*^2^ 20.7, p = 0.239; CFI = 0.976, RMSEA 0.046] of a 3 factor solution: traffic safety (3 items), traffic hazards (4 items), and a single item assessing cul-de-sacs. Mean scores of traffic safety and traffic hazards sub-scales were used in analyses.

Perceived Stranger Danger was measured by four items (e.g. “I am afraid of my child being taken or hurt by a stranger in a local park.”) from the Crime Safety sub-factor from the NEWS-Y [33], with 4-response options. The mean scale score was used in analyses.

Availability of Active Play Equipment was assessed by eight items of common free or fixed play equipment, similar to equipment assessments conducted in childcare settings [34]. A summed reversed score was used.

Places for children’s PA was assessed by a modified version of the recreation places in the neighborhood subscale of the NEWS-Y (12 items) [33]. The response option was… “within a 15-minute walk of your home?” (yes/no). Similar questionnaires have been used assessing the impact of proximity of environmental resources on children’s PA [35].

Neighborhood Informal Social Control was measured by a new scale developed for this study. The scale was informed by 2 focus groups with Latina parents using nominal group technique methods [36,37]. Items were generated by asking parents, “What sorts of things can people do to make their neighborhood safe for young children?” This resulted in development of a 19-item scale gauging parent agreement/disagreement (5-point scale) with each statement. Two items were dropped due to inadequate test-retest reliability. CFA of the 17 items showed adequate fit [Yuan-Bentler residual-based Χ^2^142.7, p = 0.053; CFI = 0.974, RMSEA 0.078] with a priori model with two subscales: political activism (7 items) and involvement (10 items). For analyses, the mean score of the full scale for neighborhood informal control was used since the two subscales were highly correlated (r = 0.73).

### Data analyses

A total of 240 Latino parents were enrolled. Eight had missing data, leaving a final sample of 232. Internal reliabilities were assessed using Cronbach’s alpha (acceptable > 0.70), or inter-item correlation (acceptable IIC > 0.20) [38] for sub-scales with few items (five or less); and test-retest reliabilities using intra-class correlations (ICC) [39]. Separate regression models evaluated each parenting practice subscale. The scores on the Hispanic and non-Hispanic acculturation sub-scales were relatively highly negatively correlated (r = -.60) and the variability of the former was low. Hence, only the non-Hispanic acculturation score was entered in the regression models. To examine the independent contributions of each set of factors on each parenting practice sub-scale, two-level mixed regression models with random intercepts of census block groups were built in a hierarchical fashion consisting of four steps: 1) socio-demographic factors; 2) cultural factors; 3) objectively-assessed environmental factors; and 4) perceived neighborhood environmental factors. After each step, the increase in total explained outcome variance (R^2^) was computed.

Given that some participants resided in the same census block groups (232 participants from 163 census block groups), thus shared common environments, two-level mixed regression models (respondents nested within census block groups) with random intercepts of census block groups were used to account for dependency in the data. Robust variance estimates were employed to address slight departures from normality or regression residuals. All analyses were conducted using Stata 12 (College Station, TX, 2011).

## Results

### PA parenting practice variance by sets of correlates

Table 2 reports the total proportion of outcome variances explained by each independent set of correlates and by all correlates in total (last column) and the statistical significance of the change in proportion of variance explained after including a specific set of correlates in the mixed regression models. Socio-demographic correlates were significantly related to having outdoor toys available for the child, psychological control, and the promotion of inactivity. Cultural factors explained a significant proportion of the variance in safety concerns. Objectively-measured environmental attributes did not contribute to the explanation of parenting practices, while perceived environmental attributes explained variability in responses on five out of seven parenting practices (engagement/structure, registering child for sports/dance, having outdoor toys available, safety concerns, and psychological control). In the combined models, the variance explained for the PA parenting practice factors ranged from 6.8–38.9%.

**Table 2:** Incremental proportion of variance in physical activity-related parenting practices explained by sets of correlates (main effects) (n = 232)

	**Set of correlates added to the regression models of parenting practices**				
**Parenting practice**	**Socio-demographic**	**Cultural**	**Objective environmental**	**Perceived environmental**	**All correlates**^**#**^
**Encouraging PA Parenting Practices**					
Engagement/Structure	0.040	0.020	0.003	0.149**	0.212***
Register child for sports or dance	0.016	0.001	0.004	0.085*	0.106**
Have outdoor toys available for child	0.045*	0.026	0.008	0.117**	0.196***
**Discouraging PA Parenting Practices**					
Safety concerns	0.043	0.046*	0.005	0.295***	0.389***
Psychological control	0.071*	0.008	0.011	0.079*	0.170***
Promote inactivity	0.074*	0.002	0.014	0.022	0.112*
Promote screen time	0.025	0.012	0.016	0.014	0.068*

Note: Data were collected in Harris County, TX from July 2011 to March 2012.

All regression analyses were conducted using mixed linear models accounting for CBG-level clustering effects. The proportion of variance represents the total amount of within- and between-CBG variance explained in parenting practices. Socio-demographic correlates include child’s and respondent’s ages, child’s gender, highest educational attainment in the household, type of home, and children in the household; Cultural correlates include acculturation (English) and familism; Objective environmental correlates are crime index, traffic index, and distance to closest park; Perceived environmental correlates are signs of physical and social disorder, traffic safety, traffic hazards, stranger danger, availability of active play equipment, places for child’s physical activity, and neighborhood informal social control.

^#^Total variance explained by all correlates. *p < .05; **p < .01; ***p < .001.

### Individual correlates contribution to PA parenting practices

Table 3 shows the adjusted associations (in the form of main effect regression coefficients) of each correlate with scores on each parenting practice factor. A few significant associations of socio-demographic characteristics with parenting practices were observed. After adjustment for other correlates, cultural and objective environmental factors were unrelated to parenting practices, with the exception of distance to the nearest park which showed a weak positive association with frequency of use of psychological control. On the other hand, parent’s perception of the neighborhood was associated with multiple PA parenting practice factors in the full models. One or more perceived environmental variables were associated with all the parenting practice factors except promotion of inactivity and screen time, for which none of the perceived environmental variables made a significant contribution.

**Table 3:** Adjusted associations (regression coefficients and 95% confidence intervals) of socio-demographic, cultural, and environmental correlates with physical activity-related parenting practices in caregivers of Hispanic-American preschool children (n = 232)

	**Physical-activity related parenting practice**						
**Correlate**	**Engagement/structure**	**Register child for sports or dance**	**Have outdoor toys available for child**	**Safety concerns**	**Psychological control**	**Promote inactivity**	**Promote screen time**
***Socio-demographic***							
Child’s gender (ref: male)							
Female	0.04 (-0.11, 0.19)	-0.15 (-0.52, 0.23)	-0.25 (-0.52, 0.02)	0.10 (0.08, 0.28)	0.13 (-0.05, 0.31)	0.07 (-0.10, 0.25)	0.01 (-0.17, 0.18)
Child’s age	-0.05 (-0.14, 0.04)	0.01 (-0.21, 0.23)	-0.10 (-0.25, 0.07)	-0.02 (-0.13, 0.09)	0.02 (-0.09, 0.13)	-0.20*** (-0.30, -0.10)	-0.01 (-0.11, 0.09)
Respondent’s age	0.01 (-0.01, 0.02)	-0.02 (-0.05, 0.02)	0.01 (-0.01, 0.04)	-0.01 (-0.02, 0.01)	-0.02* (-0.03, -0.01)	-0.01 (-0.02, 0.01)	0.02* (0.00, 0.03)
# children in household	-0.08* (-0.15, -0.02)	0.01 (-0.15, 0.17)	0.01 (-0.11, 0.13)	0.01 (-0.08, 0.08)	0.05 (-0.03, 0.12)	-0.05 (-0.12, 0.03)	0.02 (-0.05, 0.10)
Highest education in household (ref: < high or technical school)							
High or technical school	-0.15 (-0.35, 0.06)	-0.18 (0.71, 0.35)	-0.32 (-0.68, 0.05)	-0.15 (-0.40, 0.10)	-0.07 (-0.33, 0.19)	0.13 (-0.12, 0.37)	0.02 (-0.22, 0.26)
At least some college	-0.35* (-0.63, -0.06)	0.11 (-0.60, 0.82)	-0.49 (-1.01, 0.04)	-0.03 (-0.38, 0.32)	-0.15 (-0.50, 0.19)	0.01 (-0.32, 0.34)	-0.14 (-0.47, 0.18)
Type of home (ref: single family or duplex)							
Apartment/condo or other	0.12 (-0.06, 0.30)	-0.27 (-0.69, 0.14)	-0.40* (-0.74, -0.05)	0.20 (-0.03, 0.42)	0.22* (0.01, 0.42)	0.01 (-0.20, 0.21)	-0.16 (-0.36, 0.04)
***Cultural***							
Acculturation – non-Hispanic	0.08 (-0.02, 0.17)	-0.02 (-0.26, 0.21)	0.17 (-0.01, 0.34)	-0.01 (-0.12, 0.12)	-0.05 (-0.16, 0.07)	-0.08 (-0.23, 0.07)	0.09 (-0.02, 0.19)
Familism	0.09 (-0.03, 0.22)	-0.30 (-0.63, 0.03)	-0.09 (-0.32, 0.13)	-0.16 (-0.32, -0.01)	-0.02 (-0.18, 0.14)	-0.01 (-0.12, 0.10)	0.04 (-0.11, 0.19)
***Objective environmental***							
Crime index	0.001 (-0.001, 0.001)	0.001 (-0.001, 0.002)	0.001 (-0.001, 0.002)	-0.001 (-0.001, 0.001)	0.001 (-0.001, 0.001)	0.001 (-0.001, 0.001)	-0.001 (-0.001, 0.001)
Traffic index	-0.007 (-0.025, 0.011)	0.004 (-0.034, 0.043)	-0.003 (-0.040, 0.034)	0.004 (-0.019, 0.027)	0.004 (-0.014, 0.022)	0.005 (-0.015, 0.026)	0.010 (-0.010, 0.029)
Distance to nearest park (km)	0.001 (-0.02, 0.04)	0.01 (-0.06, 0.009)	0.02 (-0.05, 0.09)	-0.01 (-0.06, 0.02)	0.03* (0.00, 0.07)	0.03 (-0.01, 0.07)	0.01 (-0.02, 0.05)
***Perceived environment***							
Signs of physical and social disorder	0.24*** (0.12, 0.36)	-0.19 (-0.49, 0.10)	-0.15 (-0.38, 0.08)	0.05 (-0.10, 0.21)	-0.10 (-0.24, 0.04)	-0.07 (-0.21, 0.07)	0.07 (-0.07, 0.21)
Traffic safety	0.16* (0.03, 0.28)	0.12 (-0.19, 0.43)	0.09 (-0.14, 0.33)	-0.12 (-0.27, 0.04)	0.03 (-0.12, 0.18)	0.01 (-0.14, 0.15)	-0.12 (-0.26, 0.03)
Traffic hazards	-0.06 (-0.17, 0.05)	-0.12 (-0.40, 0.17)	0.08 (-0.13, 0.28)	0.33*** (0.20, 0.47)	0.09 (-0.04, 0.23)	0.09 (-0.04, 0.22)	-0.03 (-0.16, 0.10)
Stranger danger	0.01 (-0.08, 0.10)	-0.06 (-0.28, 0.16)	0.02 (-0.15, 0.18)	0.21*** (0.10, 0.32)	0.15** (0.04, 0.26)	0.02 (-0.08, 0.13)	-0.03 (-0.13, 0.07)
Availability of active play equipment	-0.01 (-0.02, 0.01)	0.01 (-0.02, 0.05)	0.06*** (0.03, 0.09)	-0.05*** (-0.06, -0.03)	0.01 (-0.02, 0.02)	0.01 (-0.01, 0.03)	0.00 (-0.02, 0.02)
Places for child’s physical activity	0.03* (0.00, 0.06)	0.08* (0.01, 0.15)	0.01 (-0.04, 0.07)	-0.03 (-0.06, 0.01)	-0.04* (-0.08, -0.01)	0.01 (-0.03, 0.04)	0.01 (-0.03, 0.03)
Neighborhood informal social control	0.15* (0.03, 0.27)	0.17 (-0.12, 0.46)	0.24* (0.03. 0.46)	-0.05 (-0.19, 0.10)	0.12 (-0.02, 0.26)	-0.03 (-0.17, 0.10)	0.04 (-0.10, 0.17)

Note: Data were collected in Harris County, TX from July 2011 to March 2012.

All regression analyses were conducted using mixed linear models accounting for CBG-level clustering effects. Ref = reference category; # = number. *p < .05; **p < .01; *** *p* < .0.

## Discussion

In general, parents’ perception of their neighborhood’s physical and social environment had the greatest effect on the variance of the different types of PA parenting practices, while objectively measured characteristics of the neighborhood had little to no impact on the PA parenting practices measured. Specifically, parental perceptions of perceived physical and social disorder, traffic safety, availability of places for child’s PA in the neighborhood, and presence of neighborhood social informal control were positively associated with parental engagement/structure for promoting child PA. Parents who perceive their neighborhood to have low traffic risks, plenty of places for children to engage in PA, and adequate neighborhood informal social control may feel more comfortable engaging with their child in PA. Alternatively, parents who believe it is important to engage in PA with their child, may self-select to reside in neighborhoods with such characteristics. It is not self-evident why parents who perceive more physical and social disorder in their neighborhood provided greater engagement and structure to promote PA. It is possible that parents who perceive neighborhood social disorder may feel they need to take a more active, engaging role with their child in order to provide supervision for their child.

Places for child PA was also positively associated with registering the child for sports or dance, possibly due to these locations having more opportunities for registering children in PA activities (e.g. sports or dance). Having outdoor toys available was positively associated with availability of active play equipment (as expected) and neighborhood informal social control. Parents who perceived greater traffic hazard in their neighborhood and stranger danger also reported more use of discouraging PA for safety concerns, as would be expected. The availability of active play equipment was negatively associated with safety concerns, likely due to the fact that parents make such toys available only if they believe outdoor play to be safe. Lastly, parental report of stranger danger was positively, while places for child’s PA was negatively associated with psychological control respectively. This is one of the first studies that has investigated psychological control as a practice to impact children’s PA and warrants further study to explore these findings.

In addition to parental perceptions of their neighborhood influencing their use of PA parenting practices, a few sociodemographic variables were also significant correlates. For example, parents with older children were less likely to promote inactive transport. This sub-scale included items such as carrying the child, and pushing them in a stroller instead of letting them walk, which parents are likely to do less as the child gets older. Parents who lived in an apartment were less likely to have outdoor toys available, which may be due to space limitations, but more likely to use psychological control.

Only a few other studies have investigated correlates of PA parenting practices [15,16]. One investigated child and parental characteristics as correlates of PA parenting to restrict sedentary time, monitor, and stimulate PA among 5 year old children in the KOALA birth cohort [16]. Unlike that study from Europe, we did not find that child gender was significantly associated with PA parenting practices, nor that lower education among parents was associated with less use of practices to stimulate PA. Instead, in this Latino sample, higher parental education was associated with less frequent use of Engagement/Structure for PA. The second study [15] focused on family and community factors. Their combined model explained 37% of the variance of supportive PA parenting practices among a sample of low-income, mostly non-Hispanic white or black parents of preschool-aged children. Unlike this study, community factors were not significantly associated with PA parenting practices in their multivariate models. This may be due to different instruments being used to assess PA parenting; different neighborhood characteristics assessed, and populations of different ethnicities being sampled.

Characteristics of the neighborhood where children reside have been associated with children’s PA [17-21,23], but not consistently [40,41]. The current study provides evidence that Latino parents’ perception of their neighborhood’s environment influenced how likely they were to encourage or discourage PA among their preschool-aged child, which may in part mediate the correlation of neighborhood characteristics with children’s PA. In this sample, the perceptions of their neighborhood were more influential than objectively measured neighborhood characteristics. This is contrary to a review that found objectively measured neighborhood attributes to be more consistently associated with youth’s PA than perceived neighborhood characteristics [22]. It is possible that parental perceptions of the neighborhood are more important for influencing parental behaviors to encourage or discourage PA among younger children, than older children’s PA behaviors. Youths’ PA may be more directly influenced by the objectively measured attributes, such as distance to a park.

Acculturation was correlated with PA among Latino adults, with greater acculturation associated with more PA in most Latino adult studies [42]. The associations of acculturation and PA among youth is less clear [43-47]. In the present study, cultural variables only had a weak main effect on PA parenting practices, specifically on discouraging PA due to safety concerns.

This study had several strengths including a sample at high risk for obesity who may be less active; stratified sampling to get a varied neighborhood environment exposure; assessing PA parenting practices that both encourage and discourage child PA with a validated instrument [10]; and using previously developed validated scales to assess neighborhood characteristics and cultural variables whenever possible. There were also limitations to consider. The study only sampled from one US city, and may not be representative of other Latino communities. Due to response burden we did not assess psychological variables, such as parental stress and depression, previously associated with PA parenting [15]. Only three objective neighborhood characteristics were assessed, and for the traffic and crime variables, the data were a few years old. This is a problem when relying on data that are only released intermittently and may not accurately reflect the current status of neighborhoods that have recently undergone a revitalization or decline.

## Conclusions

Primarily socio-demographic and perceived environmental variables significantly contributed to explaining 6.8–38.9% of the variance of PA parenting practices. Parental perception of their neighborhood appeared to be the most significant correlates. These findings suggest that interventions to promote PA among young children and target parents as agents of change need to consider multiple factors to effectively change parent’s behaviors to encourage and not discourage PA among their preschool aged children. This may be achieved by tailoring or adapting interventions to specific family social-ecological contexts.

## Abbreviations

CFA: Confirmatory factor analyses; CFI: Comparative Fit Index; GIS: Geographic Information System; ICC: Intra-class correlations; IIC: Inter-item correlation; NEWS-Y: Neighborhood Environment Walkability Sale for Youth; PA: Physical activity; PPAPP: Preschooler’s Physical Activity Parenting Practices; RMSEA: Root Mean Square Error of Approximation.

## Competing interests

The authors declare they have no competing interests.

## Authors’ contributions

TMO was the PI of the study, oversaw all aspects of the study protocols, data collection, interpretation of analysis, and drafted the manuscript. REL, EC, T-AC, SOH, JAM, and TB regularly attended biweekly Ninos Activos study meetings, provided input on the study protocol, helped interpret the data analyses, and critically read and edited the manuscript. REL and NP conducted the GIS mapping components of the study. EC was the primary statistician who conducted the statistical analyses, T-AC conducted some of the descriptive and CFA models. All authors read and approved the final manuscript.

## Pre-publication history

The pre-publication history for this paper can be accessed here:

http://www.biomedcentral.com/1471-2458/14/707/prepub
